# Durvalumab-Induced Hypoparathyroidism

**DOI:** 10.1016/j.aed.2025.04.005

**Published:** 2025-04-23

**Authors:** Christopher Chan, Varun Manoharan, Manimegalai Manoharan

**Affiliations:** 1 Diabetes and Endocrine Service, Liverpool Hospital, New South Wales, Australia; 2 South Western Sydney Clinical Campuses, University of New South Wales, New South Wales, Australia; 3 Concord Clinical School, The University of Sydney, New South Wales, Australia; 4 Nepean Clinical School, Faculty of Medicine and Health, The University of Sydney, Penrith, New South Wales, Australia; 5 Macarthur Clinical School, Western Sydney University, New South Wales, Australia

**Keywords:** durvalumab, hypoparathyroidism, immune checkpoint inhibitors

## Abstract

**Background/Objective:**

There have been more reported immune-related adverse events with increasing use of immune checkpoint inhibitors (ICIs) in the treatment of cancers. A range of endocrinopathies have been reported; however, ICI-induced hypoparathyroidism remains a rare immune-related adverse event. The objective of this report is to describe a rare case of durvalumab-induced hypoparathyroidism.

**Case Report:**

We describe a case of a 64-year-old man who presented to the emergency department with acute symptomatic hypocalcemia 2 years after completion of durvalumab treatment for stage III non–small cell lung cancer, who had a suppressed parathyroid hormone level, which persisted with correction of calcium levels.

**Discussion:**

ICI-induced hypoparathyroidism is a rare entity with only few cases reported in the literature, the mechanism for which remains unclear. Persistence of hypoparathyroidism despite discontinuation of immunotherapy makes inflammation and immune-mediated destruction a possible explanation.

**Conclusion:**

This case reinforces the need for an increased awareness and recognition of durvalumab-induced hypoparathyroidism, in addition to more investigation into its underlying mechanisms and potential management options.

**Highlights**

• Durvalumab-induced hypoparathyroidism is a rare but important immune-related adverse event
• Persistence of hypoparathyroidism despite cessation of checkpoint inhibitor therapy is possible
• Vigilance is needed to ensure earlier detection and management of complications including symptomatic hypocalcemia

**Clinical Relevance**

This case report highlights the rare but potential immune-related adverse events of durvalumab therapy, with increasing immunotherapy use. It is of importance to increase awareness of hypoparathyroidism as a side effect to ensure early detection and prompt management.

## Introduction

Immune checkpoint inhibitors (ICIs), such as durvalumab, which targets the programmed death-ligand 1, have shown established efficacy in treatment of a range of cancers. However, their use has exposed a discrete set of immune-related adverse events secondary to a reduction in self-tolerance.^1^ Endocrinopathies such as thyroid dysfunction, hypopituitarism, adrenocortical dysfunction, and type 1 diabetes mellitus have been more commonly reported; however, hypoparathyroidism is rare.^2^ We report a case of a 64-year-old man with hypoparathyroidism in the context of durvalumab use for lung cancer, highlighting the need for both increased vigilance of this adverse effect and more extensive research into patient risk stratification and management.

## Case Report

A 64-year-old man with stage IIIB (T3N3M0) non–small cell lung cancer (NSCLC) presented to the emergency department (ED) in September 2021 with a 5-day history of diarrhea and worsening abdominal cramping, paresthesias, and carpopedal spasm. His NSCLC involved a right upper lobe mass and contralateral para-aortic lymph nodes and had been previously treated with combination cisplatin and etoposide chemotherapy with radiotherapy to the right lung and mediastinum (66 Gy, 33#), followed by 12 months of durvalumab chemotherapy (completed August 2019).

There were no other medications causing hypocalcemia being taken, including proton-pump inhibitors, antiresorptive agents or diuretics. There was no personal history of surgery or radiotherapy to the head and neck regions, nor was there a personal or family history of autoimmune conditions. Physical examination showed a positive Trousseau sign and negative Chvostek sign, and bedside electrocardiography showed QT interval prolongation.

Laboratory investigations in the ED showed marked hypocalcemia (corrected calcium level of 1.63 mmol/L [2.10-2.60 mmol/L]), hyperphosphatemia (phosphate level of 1.94 mmol/L [0.75-1.50 mmol/L]), normomagnesemia (magnesium level of 0.74 mmol/L [0.70-1.10 mmol/L]), mild vitamin D deficiency (vitamin D level of 38 nmol/L [≥50 nmol/L]), and a suppressed parathyroid hormone (PTH) level (PTH of 1.1 pmol/L [1.3-7.6 pmol/L]) (Table). Furthermore, his blood tests showed acute renal impairment with a serum creatinine level of 219 μmol/L and an estimated glomerular filtration rate of 26 mL/min/1.73 m^2^. He was biochemically euthyroid with a serum thyroid-stimulating hormone level of 1.23 mIU/L. Notably, mild hypocalcemia of 2.03 mmol/L with a PTH at the lower limit of normal at 2.2 pmol/L coincided with the completion of durvalumab therapy 2 years prior to his presentation to ED, compared with a serum calcium level of 2.46 mmol/L at baseline prior to treatment (Fig.). This was asymptomatic and consequently went unnoticed during initial evaluations. Due to the absence of clinical symptoms or significant abnormalities, further investigations were not conducted at the time.

**Table:** Laboratory Investigations

Laboratory investigation	Baseline prior to durvalumab (August 2018)	At completion of durvalumab (August 2019)	On admission in September 2021	At discharge in September 2021
Corrected calcium (mmol/L) (reference range, 2.10-2.60 mmol/L)	2.46	2.03	1.63	2.31
Magnesium (mmol/L) (reference range, 0.70-1.10 mmol/L)	0.86	0.90	0.74	0.92
Phosphate (mmol/L) (reference range, 0.75-1.50 mmol/L)	1.11	1.34	1.94	0.64
Vitamin D level (nmol/L) (normal low, ≥50 nmol/L)	…	48	38	…
Parathyroid hormone level (pmol/L) (reference range, 2.0-6.0 pmol/L)	…	2.2	1.1	0.8
Thyroid-stimulating hormone (mIU/L) (reference range, 0.27-4.20 mIU/L)	0.71	2.63	1.23	…
Creatinine (μmol/L) (reference range, 60-110 μmol/L)	76	117	219	117
Estimated glomerular filtration rate (mL/min/1.73 m^2^)	>90	57	26	56

**Fig:**
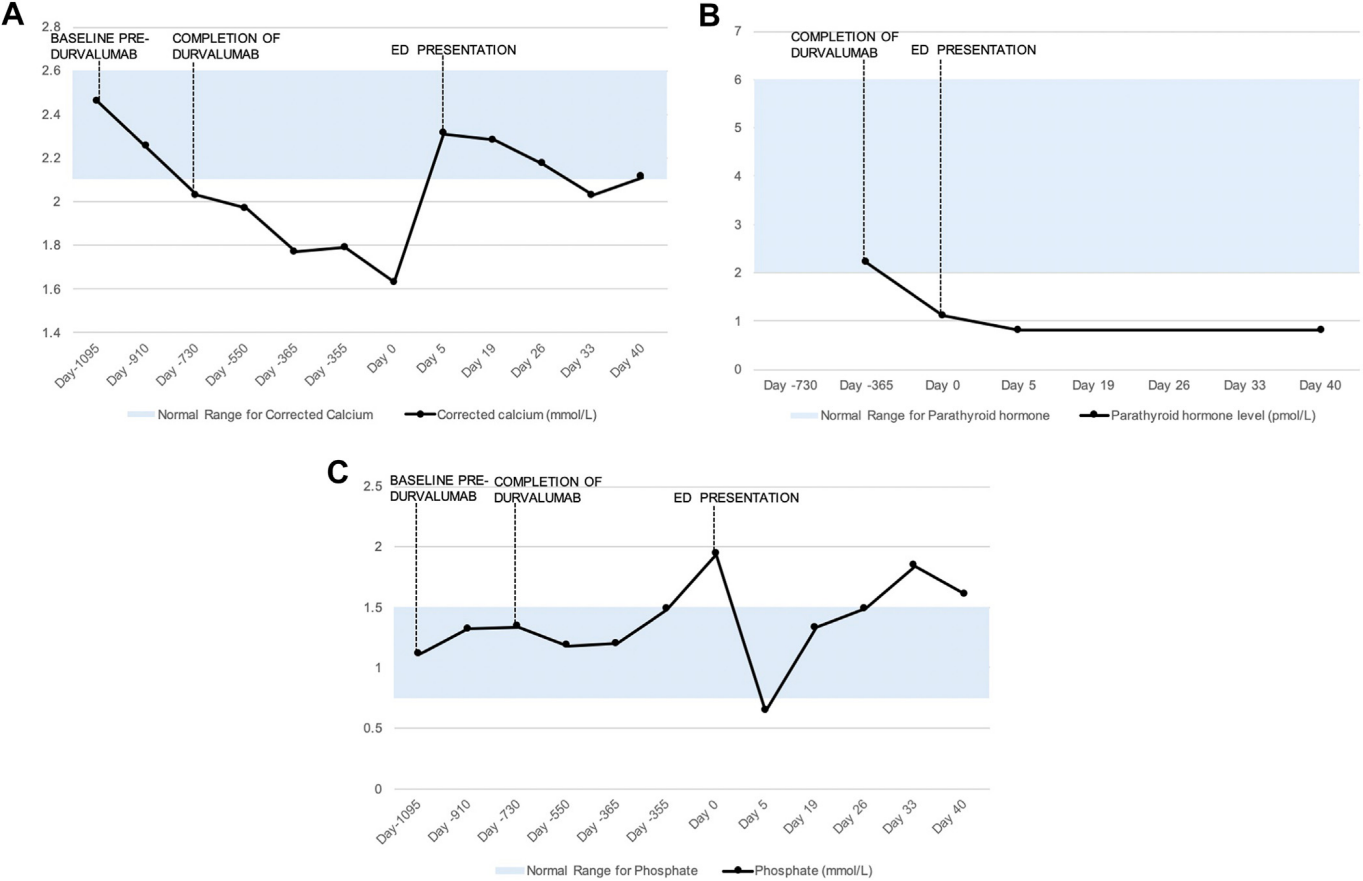
*A*, Trends in corrected calcium levels. *B*, Trends in parathyroid hormone levels. *C*, Trends in phosphate levels. *ED* = emergency department.

Repeat chest computed tomography imaging during admission had shown a stable right lung mass with no change from previous imaging after therapy.

He was admitted for treatment of acute decompensated symptomatic hypocalcemia secondary to diarrhea on a background of more long-standing hypocalcemia to a milder degree and was diagnosed with durvalumab-induced hypoparathyroidism and hypocalcemia. He was promptly managed with intravenous and oral calcium, cholecalciferol, and calcitriol. Prior to discharge, his symptoms had resolved with concomitant biochemical improvement. He was normocalcemic to 2.31 mmol/L and normophosphatemic to 1.35 mmol/L but had a persistently low PTH level at 0.8 pmol/L. He was discharged to continue oral calcium supplementation, cholecalciferol, and calcitriol, with monitoring of his serum calcium, vitamin D, and PTH levels with his general practitioner and referral to an endocrinologist for long-term management of his hypoparathyroidism and surveillance of other endocrinopathies. On serial monitoring with an endocrinologist a year after diagnosis, he remained normocalcemic to 2.50 mmol/L and normophosphatemic to 1.30 mmol/L with a lower PTH level at 0.3 pmol/L. All PTH levels were measured using the Roche assay.

## Discussion

The prevalence of hypoparathyroidism has been reported as 22 to 37 cases per 100 000 person-years consisting of both acquired and hereditary causes.^3^ Anterior neck surgery accounts for 75% of acquired hypoparathyroidism, with the remainder of cases consisting of autoimmune, radiation-induced, and infiltrative disease.^4^ ICI-induced hypoparathyroidism is an even rarer entity with only few cases of severe hypocalcemia secondary to hypoparathyroidism reported in the literature in patients treated with other ICIs including nivolumab, ipilimumab, pembrolizumab, and ipilimumab/nivolumab combination therapy.^5^, ^6^, ^7^, ^8^, ^9^ Durvalumab has been associated with ICI-induced endocrinopathies in 17% of patients in cross-sectional studies, most of which were thyroid dysfunction.^10^

In the absence of evidence of other associated causes of hypoparathyroidism, to our knowledge, this is one of the few reported cases of durvalumab-induced hypoparathyroidism in the existing literature.^11^ The patient had clinical manifestations of acute severe symptomatic hypocalcemia including worsening abdominal cramping, paresthesias, and carpopedal spasms, in which diarrhea from gastrointestinal illness caused decompensation from more chronic milder hypocalcemia. These symptoms corrected with adequate treatment with an aim for corrected calcium levels in the low-normal range because overtreatment has been associated with adverse events such as hypercalciuria and its sequelae.^12^ Ionized calcium and associated bone turnover markers were not monitored but are important adjuncts in management.

There have been reported events of durvalumab-induced hypocalcemia without confirmed hypoparathyroidism in major studies for treatment of lung cancer.^13^ The U.S. National Cancer Institute Common Terminology Criteria for Adverse Events outline degrees of severity of adverse events from chemotherapy, from mild or asymptomatic events (grade 1) to deaths secondary to events (grade 5).^14^ Hypocalcemia is graded as follows: (1) grade 1, <lower limit of normal to 2.0 mmol/L; (2) grade 2, <2.0 to 1.75 mmol/L with symptoms; (3) grade 3, <1.75 to 1.5 mmol/L where hospitalization is indicated; (4) grade 4, <1.5 mmol/L with life-threatening consequences; and (5) death with grade 5. Durvalumab-induced hypocalcemia of all grades has been reported as occurring in 46% in patients with NSCLC compared with 41% in the placebo arm in post hoc analyses from the PACIFIC study.^13^^,^^15^ In terms of more symptomatic and severe hypocalcemia, 0.2% of patients treated with durvalumab experienced grade 3 to 4 hypocalcemia compared with 0.0% in the placebo arm in these analyses.^13^^,^^15^

The mechanism of ICI-related hypoparathyroidism remains unclear; however, it is postulated that either antiparathyroid or calcium-sensing receptor–activating autoantibodies are implicated in its pathophysiology.^6^^,^^16^ Another potential explanation is of inflammation and immune-mediated destruction.^7^ Further research is needed to elucidate the role of these antibodies and whether ICI-associated hypoparathyroidism is a destructive autoimmune process similar in other endocrinopathies.

Dissimilar to other immune-related adverse events, endocrinopathies often have irreversible manifestations requiring long-term hormone replacement therapy; however, the reason behind this is still unknown.^17^ Persistence of hypoparathyroidism, despite cessation of immunotherapy, has been reported in the few case reports in the literature.^7^ Ongoing follow-up of these cases and their outcomes in addition to reporting of new cases of ICI-induced hypoparathyroidism is required to supplement the limited existing data and guide approach to management.

It is also important to note that the median time to onset of ICI-induced endocrinopathies is dependent on the type of agent and endocrinopathy, ranging from 1.4 to 11 months.^18^^,^^19^ Interestingly, the time to detected hypoparathyroidism for this case was 24 months, compared with the few case reports ranging from 1 to 15 months.^5^, ^6^, ^7^, ^8^, ^9^

Current guidelines mention the existence of ICI-related hypoparathyroidism but make no specific recommendations on screening, evaluation, and long-term management and surveillance.^20^^,^^21^

## Conclusion

Further assessment of hypocalcemia is warranted among those treated with ICIs such as durvalumab. Clinicians should be aware of hypoparathyroidism as a rare but potential adverse effect to ensure early and appropriate treatment. More extensive research is required into the mechanism behind ICI-associated hypoparathyroidism, long-term outcomes of such cases, and potential for risk stratification and management options.

## Disclosure

The authors have no conflicts of interest to disclose.
